# Spatial Autocorrelation Analysis of Land Use and Ecosystem Service Value in the Huangshui River Basin at the Grid Scale

**DOI:** 10.3390/plants11172294

**Published:** 2022-09-02

**Authors:** Feifei Shi, Bingrong Zhou, Huakun Zhou, Hao Zhang, Hongda Li, Runxiang Li, Zhuanzhuan Guo, Xiaohong Gao

**Affiliations:** 1 School of Geographical Science, Qinghai Normal University, Xining 810008, China; 2 Institute of Qinghai Meteorological Science Research, Xining 810008, China; 3 Ministry of Education Key Laboratory of Tibetan Plateau Land Surface Processes and Ecological Conservation, Xining 810008, China; 4 Qinghai Province Key Laboratory of Physical Geography and Environmental Process, Xining 810008, China; 5 Qinghai Province Key Laboratory of Disaster Prevention and Mitigation, Xining 810008, China; 6 Qinghai Provincial Key Laboratory of Restoration Ecology in Cold Regions, Northwest Institute of Plateau Biology, Chinese Academy of Sciences, Xining 810008, China; 7 Qinghai General Station of Grassland, Xining 810008, China; 8 Institute of Meteorological Development and Planning, China Meteorological Administration, Beijing 100081, China

**Keywords:** land use, ecosystem service value, GEE platform, Sentinel 1/2, Huangshui River Basin

## Abstract

The Huangshui River Basin is one of the most densely populated areas on the Qinghai–Tibet Plateau and is characterized by a high level of human activity. The contradiction between ecological protection and socioeconomic development has become increasingly prominent; determining how to achieve the balanced and coordinated development of the Huangshui River Basin is an important task. Thus, this study used the Google Earth Engine (GEE) cloud-computing platform and Sentinel-1/2 data, supplemented with an ALOS digital elevation model (ALOS DEM) and field survey data, and combined a remote sensing classification method, grid method, and ecosystem service value (ESV) evaluation method to study the spatial correlation and interaction between land use (LU) and ESV in the Huangshui River Basin. The following results were obtained: (1) on the GEE platform, Sentinel-1/2 active and passive remote sensing data, combined with the gradient tree-boosting algorithm, can efficiently produce highly accurate LU data with a spatial resolution of 10 m in the Huangshui River Basin; the overall accuracy (OA) reached 88%. (2) The total ESV in the Huangshui River Basin in 2020 was CNY 33.18 billion (USD 4867.2 million), of which woodland and grassland were the main contributors to ESV. In the Huangshui River Basin, the LU type, LU degree, and ESV have significant positive spatial correlations, with urban and agricultural areas showing an H-H agglomeration in terms of LU degree, with woodlands, grasslands, reservoirs, and wetlands showing an H-H agglomeration in terms of ESV. (3) There is a significant negative spatial correlation between the LU degree and ESV in the Huangshui River Basin, indicating that the enhancement of the LU degree in the basin could have a negative spatial spillover effect on the ESV of surrounding areas. Thus, green development should be the future direction of progress in the Huangshui River Basin, i.e., while maintaining and expanding the land for ecological protection and restoration, and the LU structure should be actively adjusted to ensure ecological security and coordinated and sustainable socioeconomic development in the Basin.

## 1. Introduction

Global and regional ecosystems are facing serious threats under the impacts of climate change and human activities [1]. A total of 60% of China’s ecosystem services have decreased as a result of the increasing pressure on the ecological environment [2]. In the context of the new era of building a global united front of ecological civilization that can actively respond to the global ecological crisis, the study of the assessment, spatiotemporal evolution, and mutual feedback relationship of land use/cover change (LUCC) and ecosystem services (ESs) has become a hotspot in the cross-disciplinary research of applied ecology, human geography, and ecological economics [3,4]. As a basic element of human social activity, land use (LU) drives the evolution of ecosystems and their service functions by changing the original surface-coverage conditions, while ESs provide the necessary environmental and physical basis for the functioning of human society; they are related to the wellbeing and regional sustainable development of humans, constitute an important element of the United Nations Millennium Ecosystem Assessment and can be calculated by and reflected in ecosystem service values (ESVs) [5]. Since Costanza, R. et al. [6] took the lead in calculating global ESVs in 1997, scholars from various countries have successively evaluated the global and regional ESVs in their various stages. Based on the Costanza assessment model, the Chinese scholars, Ouyang, Z. et al. [7] and Xie, G. et al. [8], have completed the construction of the ESV equivalent scale per unit area of China’s terrestrial ecosystems and the preliminary calculation of ESVs, which provide support for subsequent ESV assessments conducted in China. In recent years, ESV research in China has been very active and has achieved fruitful results. The research content has shifted from the initial single-ecosystem ESV calculation to the assessment methods and driving mechanisms, and the research subjects have also changed from administrative regions and typical watersheds to a more refined grid scale [4,9].

In the context of economic globalization, the urbanization process of human beings has become irreversible [10]. With the accelerating pace of urbanization in China, changes in LU patterns have triggered many ecological and environmental problems; the evolution of the spatial distribution patterns of LUCC and ESV and their response relationships have become a hot topic in the field. In the latest research results, Dai, W. et al. [11], Lei, J. et al. [12], and Xu, N. et al. [13] used spatial statistical analysis to conduct exploratory studies on the spatial correlation patterns and agglomeration patterns of LU and ESVs in eastern China’s cities; the visual expression of spatial differentiation and quantitative analysis methods that were applied hold important implications for subsequent studies. However, a literature review shows that the existing studies mainly focused on the developed eastern provinces and cities, while insufficient attention has been devoted to the underdeveloped western regions, the ecologically fragile areas of the Qinghai–Tibet Plateau, and the key areas for ecological functions [14]. As the foundation of human survival and development, the land has created a high number of ecosystem service values for human beings. With the growth of the population, the advancement of urbanization, and the development of the social economy, land use is undergoing dramatic changes [1]. In recent years, with the continuous growth of the population in the Qinghai–Tibet Plateau, the pressure on the ecological environment, which was originally fragile, has intensified. In 2020, there were 17 cities and 476 towns in the Qinghai–Tibet Plateau, including 5 towns with a population of more than 100,000 people. According to the statistics of the seventh census, the resident population in the Qinghai–Tibet Plateau was 13.134 million people, with an urbanization rate of 47.4%; the Huangshui River Basin was the most densely populated area in the Qinghai–Tibet Plateau, with a population of more than 3 million people and an urbanization rate of more than 80%, and it was also the area with the most prominent conflict between human beings and the land in the Qinghai–Tibet Plateau [15]. At present, China is at a vital stage of social development. As a historical trend of the development of human civilization, guided by the construction of ecological civilization, the latter is used to manage the relationship between human beings and the natural world. It mainly limits human activity to the parameters that the ecological environment can withstand and conducts the integrated protection and systematic management of mountains, forests, lakes, grassland, and sands [16]. As the roof of the world, the water tower of Asia, and the third pole of the Earth, the Qinghai–Tibet Plateau is China’s ecological security barrier. At present, both national and local governments are making every effort to transform the Qinghai–Tibet Plateau into a “model area for international ecological civilization” and a “demonstration area for carbon neutrality in China”. Therefore, studying the spatial patterns and interactions of the LUCC and ESV in the cities inhabiting the Qinghai–Tibet Plateau could effectively promote the synergistic progress of ecological protection and the high-quality development of the Qinghai–Tibet Plateau [17,18].

The Huangshui River Basin, located in the northeastern portion of the Qinghai–Tibet Plateau, is the seat of the economic circle of the Qinghai Provincial Capital and an important zone of ecological function; it is responsible for driving the economic development of Qinghai Province and safeguarding the ecological security barrier for the eastern region [19]. With the development of the Huangshui River Basin, there are also conflicts between economic growth, food security, and ecological protection, and how to balance the relationship between them has become a major scientific issue of concern for both local governments and researchers. In this study, the Huangshui River Basin is used as the research area. Based on the multi-source remote-sensing data under the GEE platform, the LU of the basin, mapped at a 10 m spatial resolution obtained in the year 2020, is prepared and the spatial-pattern-distribution characteristics of the LU and ESV are analyzed, using the spatial autocorrelation method. The objectives of this study are: (1) to understand the current ESV background and LU status of the Huangshui River Basin; (2) to clarify the spatial distribution pattern and spatial autocorrelation of the LU and ESV in the Huangshui River Basin; (3) to discuss the spatial dependence between the ESV and LU and its implications for basin landscape planning. The purpose of this study is to provide guidance for the standardized management of LU, as well as for the conservation and restoration of ecosystem service values in the Qinghai–Tibet Plateau.

## 2. Overview of the Study Area

**Figure 1:**
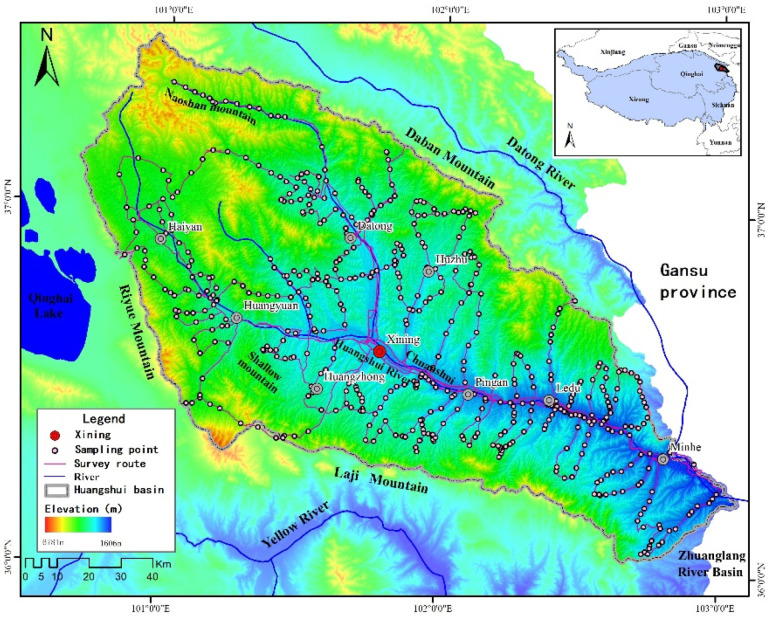
Study area and sampling site distribution; the ALOS DEM collected from the NASA Earthdata Search (https://search.asf.alaska.edu/, accessed on 1 July 2022), which was produced in October 2015.

The Qinghai Huangshui River Basin is located in the northeastern corner of the Qinghai–Tibet Plateau and belongs to the transition zone between the Qinghai–Tibet Plateau and the Loess Plateau (36°–37° N, 100°–103° E), with an area of 1.6 × 10^4^ km^2^ (Figure 1). In the west, the basin reaches Riyue Mountain and is adjacent to Qinghai Lake; in the east, it connects to the Zhuanglang River Basin in Gansu Province; in the south and north are the Laji and Daban Mountains, respectively. The Basin is surrounded by high hills. As the largest, first-order tributary of the upper reaches of the Yellow River, the main river course and tributaries of the Huangshui River run through the Basin from west to east, forming a plume-like shape; due to the long-term erosion caused by water, a geomorphological pattern of alternating canyons and basins has gradually formed that is similar to a string of pearls [20]. The terrain of the basin is high in the west and low in the east, and the elevation ranges from 1655–4860 m. According to the differences in the topography, altitude, climate, and agricultural production, the basin is naturally divided into three ecological regions, comprising river valley plains (below 2600 m), loess hills (2600–3200 m), and medium-height and tall mountains (above 3200 m) [20], which the local residents call Chuanshui, or shallow mountains, and Naoshan mountains, respectively. In the Huangshui River Basin, the climate is arid and semiarid continental, according to the statistics from 40 years of meteorological observation data derived from 9 meteorological stations in the Basin; the average temperature in summer is 12.1 to 19.7 °C; the average temperature in winter is −13.5 to −6.2 °C; the average precipitation is 329.6–537.8 mm; the average evaporation is 1188.0–1847.8 mm; the natural landscape is rich, and the vegetation types are diverse [20]. At present, the administrative region of the Huangshui River Basin includes nine counties (cities), i.e., Xining, Huangyuan, Datong, Huangzhong, Haiyan, Huzhu, Minhe, Ledu, and Ping’an. The population is approximately 3.38 million people, according to the Qinghai Provincial Statistical Yearbook 2021 [21], accounting for 57% of the province’s total population, while the gross domestic product (GDP) accounts for 64% of the province’s GDP. Therefore, the Huangshui River Basin is not only the core area of socioeconomic development in Qinghai Province but also an important display window and area of radiation for the construction of ecological civilization in Qinghai Province.

## 3. Data Sources and Research Methods

**Figure 2:**
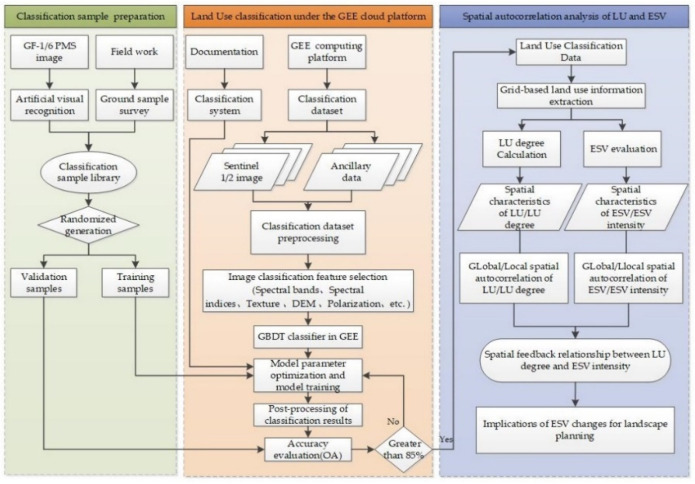
Research Procedure.

In the current study, we used the GEE cloud computing platform, adopted the Sentinel-1/2 active and passive remote-sensing images and other auxiliary data, and used the GBDT ensemble learning classification method to obtain the LU data for the Huangshui River Basin in 2020, with a spatial resolution of 10 m. With the support of high classification accuracy and high-resolution LU data, the grid analysis method, ESV evaluation method, global/local spatial autocorrelation method, and bivariate spatial autocorrelation method were used to explore the spatial-distribution characteristics and spatial-association patterns of LU and ESVs in the basin. The research framework of this paper consisted of 3 sections (Figure 2): (1) the construction of a classification sample library, based on field surveys and high-scoring 1/6 images (2 m spatial resolution); (2) LU classification under the GEE platform; and (3) a spatial autocorrelation analysis of LU and ESVs.

### 3.1. Data Sources and Preprocessing

The LU data obtained for the basin in 2020 were obtained through the automatic classification of satellite remote sensing images and manual auxiliary correction. The data sources used mainly included Sentinel-1 SAR GRD, Sentinel-2 MSI, and ALOS 12.5-meter resolution digital elevation data. The remote sensing data were directly used on the Google Earth Engine (GEE) platform, while the ALOS digital elevation model (ALOS DEM) was obtained via the NASA Earthdata Search (https://search.asf.alaska.edu/, accessed on 1 July 2022), which was produced in October 2015. [22,23]. GEE is a data-processing platform driven by Google Cloud Computing, which contains petabyte-scale remote-sensing images and geoscientific datasets. Therefore, GEE provides a powerful tool for data-driven scientific research and has been widely used to monitor the LUCC [24].

The data were screened on the GEE platform, based on the basin area, imaging time, and cloud cover. Preprocessing, including cloud masking, data resampling, and image collection by median composition and cropping, was performed on the qualified Sentinel-2 MSI time-series images from June to September 2020, while for the Sentinel-1 SAR images, which were based on different viewing angles and polarization methods, preprocessing included data classification and image collection via median composition and cropping.

### 3.2. Research Methods

#### 3.2.1. Classification System and Classification Method

Based on the field survey results for LU in the basin and by referring to the remote-sensing mapping LU classification system for China described by Liu, J. et al. [25], the land-surface types in the basin were divided into 6 first-level classes, i.e., cropland, woodland, grassland, water, urban and rural industrial/mining/residential land (referred to as urban land), and unutilized land. To improve the refinement of the classification results and highlight the LU characteristics in the basin, some LU types were subdivided into second-level classes. The grassland was subdivided into high-, medium-, and low-coverage grasslands; the woodland was subdivided into forestland and other forestland, forestland mainly included natural forests and large-scale planted forests, while other forestland mainly included shrubs, sparse forests, and tree nurseries. In addition, due to the significant ecological value of wetlands, the second-level class of marsh wetlands in the unutilized land was listed separately. Altogether, there were 10 LU types in the basin used in this study.

Before the computer-based automatic classification of LU could be performed, it was necessary to first establish a classification sample set and determine the classification feature parameters. When establishing the sample set, data obtained from 530 field sampling sites were used; 12,000 sampling sites were visually selected, based on the Sentinel-2 MSI images and GaoFen 1/6 images with 2 m spatial resolution, and a stratified random-sampling method was used to divide the samples into training and validation samples, at a ratio of 7:3. Based on the Sentinel-1/2 and ALOS DEM data, the feature parameters, such as spectral bands, spectral indices, polarization bands, texture, and terrain information, were selected on the GEE platform, as shown in Table 1. At present, GEE provides a variety of machine-learning classification algorithms. In this paper, a gradient tree-boosting ensemble learning classifier was used. This model connects multiple weak classifiers in series and each classifier learns, based on the training results of the previous model, thus ensuring a high goodness-of-fit by the classifier [26,27]. After completing the sample selection and determining the feature parameters, gradient-tree boosting was used to classify the LU in the basin, then the accuracy was verified. The confusion matrix-based overall accuracy (OA), kappa coefficient (K), producer accuracy (PA), and user accuracy (UA) were used to conduct the evaluation [28,29]. Finally, to ensure the accuracy of the classification results, obviously misclassified areas in the automatic classification results were manually corrected, based on the 2020 remote sensing images.

#### 3.2.2. The Grid Processing Method of LU

The grid is an effective evaluation unit for measuring the temporal and spatial evolutions of LU at the micro-scale and an important means of studying the spatial differentiation pattern of LU. After considering the actual cell size of the basin surface, the spatial heterogeneity of the land surface, and the computational efficiency used in similar studies, a 1 × 1 km grid was selected for this study. The basin was divided into 18,790 grid cells using the “create fishnet” tool in ArcGIS 10.7 software, and the number, area, and area ratio of each LU type in each grid were determined.

#### 3.2.3. The Grid Processing Method of LU Degree

The degree of LU reflects the degree of interference of human activities, to a certain extent. According to the natural equilibrium state of the natural land complex that is under the influence of social factors, the LU degree of each land-surface type was classified, and any land-surface type with a high degree of human disturbance was assigned a high value. In this study, the gridded LU data were used to calculate the LU degree, according to the method used by Zhuang, D. et al. [37], with Equation (1):

(1) $$L_{j}=\overset{n}{\underset{i=1}{\sum }}\frac{A_{ij}\times P_{i}}{S_{j}}$$

where *L_j_* is the LU degree index of the *j*th grid cell, *A_ij_* is the area of the *i*th LU type in the *j*th grid cell, *S_j_* is the area of the *j*th grid cell, and *n* is the number of LU types. *P_i_* is the LU degree index of the *i*th type, with urban land assigned a value of 4; cropland is assigned a value of 3; water, grassland, woodland, and wetland is assigned a value of 2; and unutilized land is assigned a value of 1.

#### 3.2.4. Grid-Based Estimation of the ESV

The ESV included the value provided by four types of supplying, regulating, supporting, and cultural services. Based on recent studies of the ESV [38,39], this study developed a table of ESV equivalent per unit area after combining the ESV equivalent per unit area that was revised by Xie, G. et al. [39] with the actual distribution of LU types in the basin (Table 2). Because coniferous forests, coniferous and broadleaf mixed forests, and broadleaf forests are found in the basin, the average ESV equivalent of these forestlands was used as the ESV equivalent of forestland, and the ESV equivalent of shrub forest was used as the ESV equivalent of the other types of forestlands. The base ESV equivalent of high-coverage grassland was based on the mean ESV equivalent of alpine grassland, alpine meadow, and scrub, while 85% and 65% of the base ESV equivalent of high-coverage grassland was used for medium- and low-coverage grasslands, respectively [40]. The mean ESV equivalent of desert and bare land was used as the base ESV equivalent of unutilized land, while the ESV equivalent of urban land was considered as 0. In this study, a direct market approach based on the correction coefficient of the value equivalent factor was used for calculating the ESV. Xie, G. et al. [39] determined that the economic value per unit of ESV equivalent in China was 3406.50 CNY/ha, and the grain yield per unit area in the Huangshui River Basin in 2020 was 3411 kg/ha, while the national grain yield per unit area was 5734 kg/ha; therefore, the correction coefficient of the ESV equivalent in the basin was 0.60, while the economic value per unit ESV equivalent in the basin was determined to be 2026.4 CNY/ha [8,21]. 

The ESV calculation method, using the gridded LU data, is presented in Equations (2) and (3):

(2) $$ESV_{j}=\overset{n}{\underset{i=1}{\sum }}A_{ij}\times C_{i}$$

(3) $$C_{i}=\overset{f}{\underset{k=1}{\sum }}EC_{k}\times E_{esv}$$

where $ESV_{j}$ is the ESV of the *j*th grid cell, $A_{ij}$ is the area of the *i*th LU type in the *j*th grid cell, $C_{i}$ is the ecological value coefficient of the *i*th LU type, *n* is the number of LU types, $EC_{k}$ is the *k*th ESV equivalent of the *i*th LU type, and $E_{esv}$ is the economic value per unit ESV equivalent, i.e., 2026.38 CNY/ha.

When calculating the ESV, the grid cells at the edge of the basin were crossed by the boundary, so they were not 1 × 1 km. Using the ESV intensity proposed by Lei, J.R. et al. [9] can better solve the problem of the underestimation of ESV in the grid cells at the edge of the basin, as presented in Equation (4):

(4) $$V_{esv_{j}}=\frac{ESV_{j}}{S_{j}}$$

where $V_{esv_{j}}$ represents the ESV intensity of the *j*th grid cell and $S_{j}$ represents the area of the *j*th grid cell.

#### 3.2.5. Spatial Analysis of the Mutual Feedback Relationship between LU and ESV

Spatial autocorrelation means that the closer specific things or phenomena are to each other in terms of their spatial position, the more similar they are [41]. That is, things or phenomena are dependent on spatial position. Spatial autocorrelation analysis is used to study the degree of spatial autocorrelation between a spatial unit and its surrounding units, using certain statistical methods and analyzing the characteristics of the spatial distribution of spatial units [41]. Following the spatial autocorrelation theory in GIS spatial statistical analysis, the pattern of the spatial correlation of LU and ESV in the basin was explored using global spatial autocorrelation (GSA), Anselin local spatial autocorrelation (LISA), and bivariate spatial autocorrelation (BSA) [41], and the analysis was performed using GeoDa 1.7 software (Dr. Luc Anselin and his team, Chicago, IL, USA). In GSA, the global Moran’s *I* was selected to characterize the spatial-distribution characteristics and degree of correlation of LU and ESV in the basin, and in a local index of spatial association (LISA), the local Moran’s *I* was selected to characterize the local spatial agglomeration and differentiation characteristics of LU and ESV in each grid cell in the basin, while the BSA mainly revealed the spatial correlation between LU and ESV in each grid cell in the basin [41].

In addition, an analysis of variance (ANOVA) was used to perform spatial-structure analysis and simulate the optimization of ESV intensity, while the ordinary kriging method was used to perform spatial interpolation and the continuous expression of ESV intensity. Specifically, after logarithmic transformation by GS + 10 software, the ESV intensity data were distributed normally. The semivariance function was then calculated and parameters such as the nugget, sill, and range were selected to describe ESV characteristics in the space, including the spatial-differentiation degree, distribution pattern, and composition [42]. Finally, the spatial data interpolation of ESV was performed using ordinary kriging in the ArcGIS 10.7 software (ESRI Inc., Redlands, CA, USA).

## 4. Results and Analysis

### 4.1. LU Classification Accuracy and Distribution Characteristics

The accuracy of the LU classification results in the basin was evaluated. The OA reached 88.06%, and the K coefficient was 0.86, indicating that the OA of the classification results was relatively high. The highest accuracies were obtained for urban land and water, with PA values of 94.57% and 94.82%, respectively. The PA for cropland, wetland, and unutilized land presented high values, ranging from 90.16–91.30%, while the PA of the low-coverage grassland reached 80.50–87.50%.

**Figure 3:**
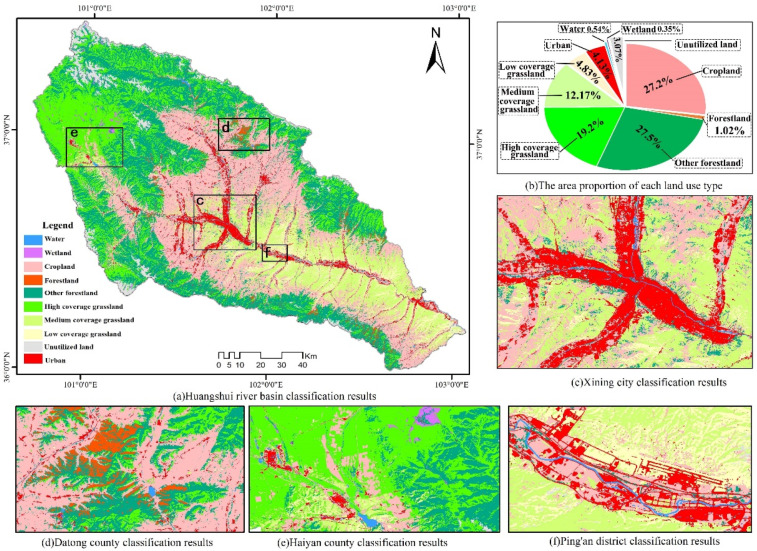
LU classification results: (**a**) the classification results for the Huangshui River Basin; (**b**) the area proportion of each LU type; (**c**) the classification results for Xining City; (**d**) the classification results for Datong County; (**e**) the classification results for Haiyan County; (**f**) the classification results for Ping’an County.

The analysis of the spatial distribution pattern and the area ratios of LU types in the basin showed that the urban land was mainly distributed on the natural terraces on both sides of the Huangshui River, accounting for approximately 4.13% of the basin area (Figure 3). The cropland was mainly distributed in the river valley plains around towns, the gentle slopes of the loess hills, and the flat land between the mountains, accounting for approximately 27.20% of the basin area. High-, medium-, and low-coverage grasslands accounted for 36.2% of the basin area, of which large extents of medium- and low-coverage grasslands were present in the transitional areas between the river valley plains and loess hills around the towns, while high-coverage grassland was mainly distributed in the areas between forests and forest margins on the medium-height mountains and dominated by natural subalpine grasslands (meadows) [43]. The woodland accounted for 28.51% of the basin area, of which the area of mature woodland was small and dominated by natural forest trees that were distributed on the shady slopes of the medium-height mountains of the basin, while other woodland was dominated by shrubland, which is a widely distributed ecological landscape type in the basin. The unutilized land was mainly observed on the bare rock and gravel slopes on the tops of mountains, the transition zones between the valley plains and the hills, and the areas of bare soil formed by erosion or artificial excavation, accounting for 3.07% of the basin area. The wetland area was small and was only distributed in the low-lying areas of Haiyan County in the northwestern portion of the basin.

### 4.2. Characteristics of LU Spatial Patterns

#### 4.2.1. Analysis of the Spatial Autocorrelation of LU

GSA reveals the overall trend of spatial autocorrelation of LU or ESV in the whole basin by calculating the global Moran’s *I* index. The global Moran’s *I* is a global assessment of spatial autocorrelation, taking values between 1 and −1; positive values indicate that land-surface types with similar attributes are clustered in space, and the greater the value, the stronger the spatial clustering. Negative values indicate that land-surface types with different attributes are clustered in space; the smaller the value, the greater the spatial heterogeneity. Values of 0 indicate that land-surface types are randomly distributed in space [41]. The global Moran’s *I* index exceeded 0 for each LU type in the basin (Table 3), and each LU type presented significant spatial autocorrelation results (*p* < 0.001). Cropland was generally distributed on a large scale, and its spatial autocorrelation was the strongest (the Moran’s *I* index reached 0.89), while wetlands and water were relatively scattered, and their spatial autocorrelations were the weakest. The Moran’s *I* index values of the other seven LU types were between 0.65 and 0.87, which were relatively concentrated in terms of spatial distribution and presented strong spatial autocorrelation values.

**Figure 4:**
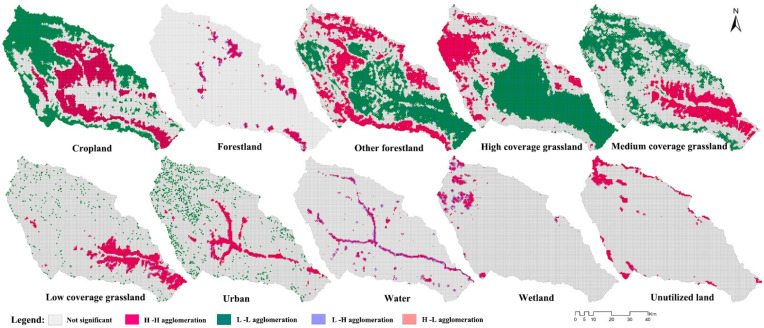
Distribution of each LU type, visualized by LISA.

LISA reflects the local characteristics of the spatial autocorrelation of LU or ESV in the basin by calculating the local Moran’s *I* index. If the index is greater than 0, this indicates that the LU or ESV value of the spatial unit is clustered as H-H or L-L, while if the index is less than 0, it indicates that the LU or ESV value of the spatial unit is clustered as H-L or L-H. H indicates that the data attribute value is higher than the average value, L indicates that the data attribute value is lower than the average value, and H-H clustering and L-L clustering indicate that the difference between the region and its surrounding areas is small; that is, the region where higher or lower values are concentrated. LH clustering and HL clustering indicate that there are differences in variable values between the region and its surrounding areas [41]. The local spatial differentiation and agglomeration characteristics of various LU types were obtained by LISA (Figure 4). Cropland showed significant H-H agglomeration in the shallow mountainous area, forestland showed H-H agglomeration in the national forest park, and other forestland showed contiguous H-H agglomeration in high mountainous areas but showed L-L agglomerations in shallow mountainous and river-valley-plain areas. High-coverage grassland and wetlands displayed H-H agglomerations in the northwest high mountainous area, while medium- and low-coverage grasslands showed H-H agglomeration in the transition zone from urban land to hilly sloping farmland. Urban land showed spatial H-H agglomeration within the X-shaped river valley, with the city of Xining. Water showed spatial H-H agglomeration along the Y-shaped river network. The unutilized land showed H-H agglomeration in the stony alpine areas (the Laji and Daban Mountains).

#### 4.2.2. Analysis of the Spatial Autocorrelation of LU Degree

**Figure 5:**
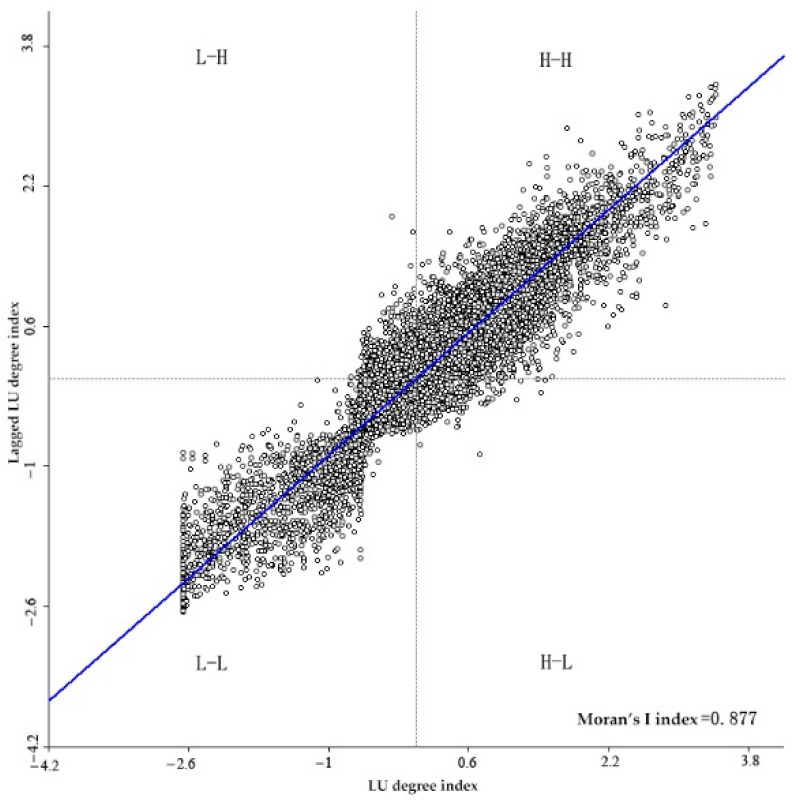
Moran scatterplot of LU degree.

**Figure 6:**
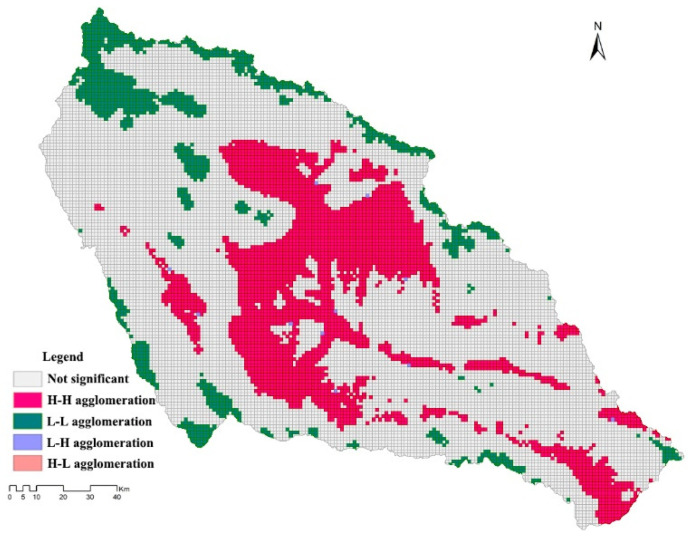
Distribution map for LU degree, visualized by LISA.

The analysis of the spatial autocorrelation of the gridded LU degree in the basin showed that the global Moran’s *I* index was 0.88 and the scattered points were mainly distributed in the H-H and L-L quadrants (Figure 5), which indicated that the LU degree in the basin exhibited a strong positive spatial correlation. The LISA analysis (Figure 6) showed that the expansion of LU, population agglomeration, and cultivated land area in Xining City and the surrounding important towns led to a high LU degree in shallow-mountain areas and high spatial concentration (H-H aggregation), while the LU degree of the unutilized land in the basin displayed an obvious L-L agglomeration.

### 4.3. Characteristics of ESV Spatial Pattern

#### 4.3.1. Analysis of ESV for Each LU Type

Table 4 presents the ESV of each LU type in the Huangshui River Basin in 2020. The total ESV of the basin reached CNY 33.18 billion (USD 4867.2 million), accounting for 11.19% of the total economic production for Qinghai Province in that year. Among the LU types, woodland exhibited the highest ESV, accounting for 43.20% of the basin ESV, and grassland presented the next highest ESV, accounting for 37.49% of the basin ESV, therefore woodland and grassland were the main contributors to the basin ESV. Although water and wetland made up less than 1% of the land use, their ESV was 8.41%, because water and wetlands had the highest ESV per unit area.

#### 4.3.2. ESV Spatial Autocorrelation Analysis

**Figure 7:**
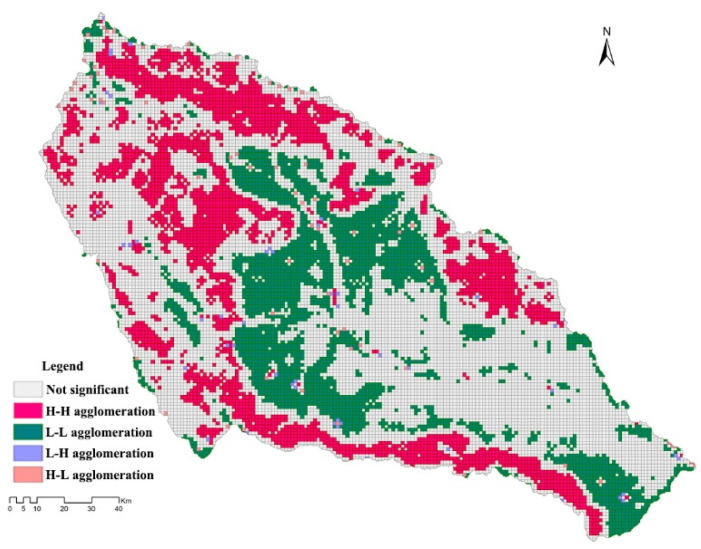
Distribution of ESV intensity, visualized by LISA.

The analysis of the spatial correlation of gridded ESV intensity in the basin showed that the global Moran’s *I* index of the basin ESV intensity was 0.65, showing a strong positive spatial correlation. Meanwhile, LISA analysis (Figure 7) showed that the ESV intensity of woodland, high-coverage grassland, reservoirs, and wetland areas presented obvious H-H agglomerations, which may be related to the rich biodiversity and ecological functions within their ecosystems. However, the ESV intensity in sloping farmland areas on the shallow hills in the areas surrounding Xining, such as parts of Datong County, Huanzhong County, and Huangyuan County, was weak and showed an L-L agglomeration in spatial terms that was affected by human activities, such as urban development, disrupting the ecological process and balance within the original system, which can easily lead to the weakening or even degradation of ESV.

**Figure 8:**
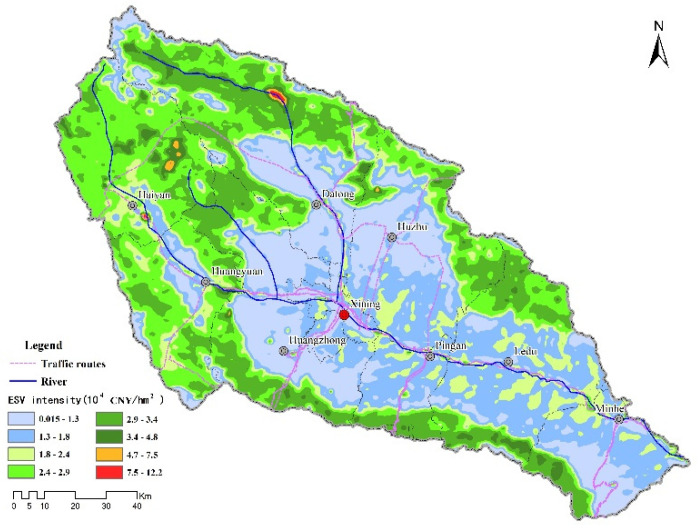
Spatial interpolation of ESV intensity.

The basin ESV intensity data after logarithmic transformation conformed to a normal distribution, according to ANOVA, and a spherical model was selected with a coefficient of determination (R^2^) of 0.92 and a residual sum of squares (RSS) of 9.57 × 10^−5^, indicating a good fit to the model. The pattern of the spatial structure differentiation of ESV intensity was analyzed using the parameters obtained by the spherical model, and the nugget and nugget effect indicated the magnitude of variation in ESV intensity, caused by random factors [42]. The nugget effect was below 25%, which indicated that the basin ESV intensity presented a strong spatial autocorrelation; the range was 5.5 km, which indicated that the basin ESV intensity had a strong spatial correlation within a grid spacing of 1 km. To more intuitively analyze the continuous spatial distribution and trends in ESV intensity, the ordinary kriging method was used to perform a spatial interpolation of the gridded ESV intensity in the basin (Figure 8); the results obtained following interpolation were highly consistent with the pattern of ESV intensity distribution obtained by LISA, in which the reservoir and wetland areas presented the highest ESV intensity due to their prominent water-conservation function. Woodland and grassland ecosystems were characterized by high ESV intensities, while cropland in shallow mountainous areas, urban agglomerations distributed along river valleys, and scattered townships were characterized by low ESV intensities.

### 4.4. Spatial Feedback Relationship between LU Degree and ESV Intensity

**Figure 9:**
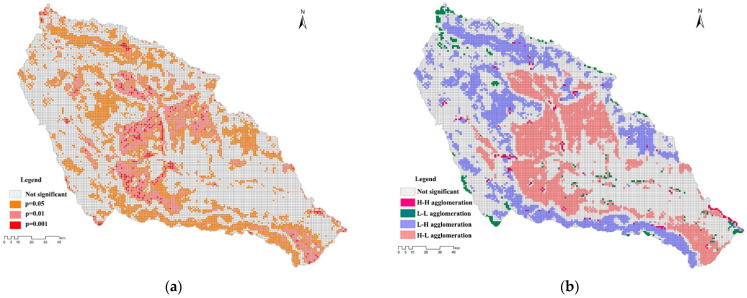
The distribution map: (**a**) LU degree and ESV intensity shown by bivariate LISA; (**b**) significance levels of LU degree and ESV intensity.

We used the global bivariate Moran’s *I* index and the local bivariate Moran’s *I* index for BSA analysis to study the degree of spatial correlation between LU degree and ESV intensity across the basin, as well as local space, respectively. In terms of the spatial interaction between the LU degree and ESV intensity in the basin, the BSA analysis showed that the global Moran’s *I* index was −0.43, which indicated that there was a significant negative spatial correlation between the two; that is, the ESV intensity tended to decrease with the increasing degree of LU. The bivariate LISA analysis demonstrated that the LU degree and ESV intensity mainly exhibited H-L and L-H agglomeration patterns in space (Figure 9a). H-L agglomeration was mainly distributed in the counties surrounding Xining important townships and cultivated land areas, showing an extremely significant correlation (*p* < 0.01); this indicates that the high LU degree and the low ESV intensity in this area formed a spatial agglomeration (Figure 9b). L-H agglomeration occurred mainly in woodland and in medium- and high-coverage grasslands, showing a significant correlation (*p* < 0.05) (Figure 9b); this indicates that the high LU degree and the low ESV intensity in this area formed a spatial agglomeration. Artificial wetland parks and small artificial lakes in the city, pits and ponds excavated in farming areas, and small reservoirs showed spatial H-H agglomerations that were sporadically distributed, demonstrating that the artificial ecological restoration projects that were conducted in Xining City played a role in alleviating the pressure of human beings on urban ecosystems and increasing the ESV. Some bare-rock and bare-soil areas presented L-L agglomerations (Figure 9a).

## 5. Discussion

### 5.1. LU Classification and Gridding Method

At present, the widespread use of the GEE cloud computing platform not only saves time in data downloading and preprocessing but also frees researchers from constraints on local computing power, thus making LUCC information acquisition more convenient and efficient [44]. The LU data used in the existing studies are still mainly at a spatial resolution of 30 m, which results in insufficient data accuracy for some areas with complex terrain and diversified surface-feature types. In this study, to obtain LU data with a high spatial resolution and high classification accuracy, the Sentinel-1/2 active and passive satellite remote-sensing data in GEE were used to obtain the basin LU information, for which the OA and K coefficients were 88% and 0.86, respectively. Compared with similar studies, the proposed method improves the accuracy and efficiency of LU classification in mountainous areas [45,46]; according to the accuracy assessment, it can be observed that this 10-meter spatial resolution LU data for the Huangshui River Basin is reliable and accurate and that the LU classification framework for mountainous areas under the GEE platform is effective. In this study, 20 kinds of remote sensing classification data were used, although these data have been widely used in similar studies and the use of all the data does not cause the classification time to be extended in the GEE platform [24]; in fact, the degree of contribution of each set of classification data to the classification accuracy is different, and the optimization of the classification data can be achieved through the feature importance assessment method to obtain greater classification efficiency. Considering the problem of surface patch fragmentation and high spatial heterogeneity in the Huangshui River Basin, the fine grid of 1 × 1 km selected in this study can better characterize the pattern of the spatial autocorrelation of the LU structure in mountainous areas (mainly the middle-high mountains and loess hills) than can the 3 × 3 km grid widely used in similar studies. Although the use of a finer grid will increase the calculation time and consume more computing resources, it can more accurately reflect the fragmented LU structure information for the mountainous terrain. At present, the grid-evaluation scale has significantly improved the visualization effect of evaluation elements, such as LU and ESV, and has broken the research paradigm that takes administrative districts as the evaluation units. In recent years, scholars have tried to use grid analysis methods and adopted finer grids, all of which have achieved better display effects for LU and ESV spatial distribution characteristics [12,40]. However, it should be pointed out that the spatial characteristics of LU and ESV are multidimensional, complex, and uncertain. Therefore, the research should focus on clarifying the spatial autocorrelation between LU and ESV, considering the spatial pattern scale effect, interpreted by the grid analysis method, and constructing the evaluation method of the optimal grid scale in different research areas. At present, what needs to be widely recognized is that Xie, Y. et al. [47] proposed a model-agnostic spatial transformation and moderation framework that can simultaneously learn the arbitrarily shaped space-partitioning of heterogeneous processes along with a “spatialized” network architecture and generalize these learned spatial structures to new regions. By using the spatial heterogeneity identified from the data and automatically adjusting the grid size according to need, the framework can properly solve the problem of grid size selection in different research areas and significantly improve the performance of base networks regarding spatial problems [47]. The concept has a bright future for promotion and application.

### 5.2. Patterns of Spatial Distribution of LU and ESV

LUCC is a dynamic process that is determined by comprehensive interactions between humans and the environment; its driving factors and mechanisms are complex and diverse and can cause spatial agglomeration and regional differences in LU [48]. Verburg, P. et al. [49] and Lei, J. et al. [12] believed that natural ecological environmental factors play a leading role in LUCC and its distribution pattern, while social factors often play a leading role in the quantitative characteristics of LUCC. In the middle-height and high mountains in the upper reaches of the Huangshui River Basin, there is little human disturbance and the water supply and temperature are suitable, so this region remains dominated by the original natural ecological landscapes, such as woodlands, alpine meadow grasslands, and wetlands. The climatic conditions in the shallow hilly areas dominated by the loess hills and low mountains are relatively favorable, but the original vegetation has gradually evolved into agricultural areas due to the powerful impact of human activities. Cities and towns are built in river valleys that offer a suitable climate and convenient transportation. With the implementation of certain strategies, such as the China Western Development and Lan-Xi Cluster Construction strategies, the aggregation of population and urban expansion has been strengthened, the original LU structure of the basin has been changed, and a contiguous area with a high LU degree and H-H agglomeration has formed, with Xining as the center radiating out to the surrounding areas. The current LU pattern in the Huangshui River Basin is closely related to its natural and social environment and shows a high spatial autocorrelation, which is consistent with the conclusions drawn by Verburg, P. et al. [49].

Using the modified value equivalent factor method, this study found that the ESV of the Huangshui River Basin in 2020 accounted for one-tenth of the total production of Qinghai Province in that year, confirming the finding that the ESV that was created in this basin, which provides the land for regional survival and development, cannot be ignored. The basin ESV exhibits a high degree of spatial autocorrelation and the ESV is mainly derived from the water conservation areas, such as woodland and grassland, indicating that the forest and grassland are key to regulating ecosystem services in the basin [50]. The water bodies in the basin perform a significant water-conservation function, with the highest ESV per unit area. However, current human activities have seriously changed the path of part of the water cycle in the Huangshui Basin. In the past 45 years, the average runoff in the basin has decreased, with a change rate of −10 million m^3^/10 a. Human activities play a leading role in this reduction in runoff, and its contribution rate has reached 64.54% [51]. In the future, more natural ecological landscape belts that integrate the purification of rivers, wetlands, and water bodies should be constructed to improve the hydrological and ecological benefits of the basin. Towns and farming areas with high-intensity social and economic activities are low ESV areas with an L-L agglomeration in space. While creating economic benefits, these areas also present challenges for the process of achieving green development in the basin.

### 5.3. Response of ESV to LU and Implications for Landscape Planning

Many studies have confirmed that the LU degree is negatively correlated with ESV, and significant spatial autocorrelation and spillover are present [12,52,53]. The spatial spillover effect specifically refers to the tendency for an increase in LU degree within an area to lead to degradation in the ESV in the neighboring areas [54]. This study observed a significant negative spatial correlation between the LU degree and ESV in the Huangshui River Basin; this increase in the basin LU degree would be expected to have a negative spatial spillover effect on the ecosystems in the surrounding areas. For example, the LISA analysis showed that areas with a high LU degree, such as cropland and urban areas, were the areas with a low ESV, while areas with a low LU degree, such as woodland, water, and wetland, were the areas with a high ESV. To achieve green development in the Huangshui River Basin in the future, in addition to maintaining the existing surface-landscape types and patterns, it is necessary to protect and gradually expand beneficial ecological landscape types, such as woodlands, grasslands, waters, and wetlands, and optimize and control the scale of arable land and construction land, to guide the development of LU structure in the direction of increasing ESV. It should be noted that the spatial characteristics of the value of LU and ecosystem services are multidimensional and complex, and the use of a single spatial statistical analysis method is still insufficient. At present, certain geospatial techniques with interdisciplinary foundations (i.e., mathematics, statistics, and computer science) have emerged; Xie, Y. et al. [55] presented case studies for five geospatial technologies; namely, hotspot detection, colocation detection, prediction, outlier detection, and teleconnection detection. In later studies, the above geospatial techniques will be able to better discover the current correlation patterns of LU and ESV space.

The Huangshui River Basin is a key area for the economic development of Qinghai Province and also the pilot area for the construction of ecological civilization in Qinghai Province [56]. After exploring the patterns of spatial distribution of LU and ESV in the basin and weighing the spatial feedback relationship between them, this study concluded that areas in the basin with low LU degrees and high ESVs should be treated as ecological core areas. For water-conservation areas, such as woodlands and wetlands, buffer zones should be designated for their strict protection, after considering the spatial proximity effect. In natural grasslands, the principle of forage–livestock balance should be followed and measures, such as rotational grazing, grazing bans, and replanting, should be adopted to curb grassland degradation. Areas with high LU degrees and low ESVs should be treated as green development areas, while for the eastern clusters of cities and towns with Xining as the center, the path of green development should be explored by actively adjusting the LU structure and industrial layout to create additional ecological landscapes, such as urban greenspace, ecological corridors, and riverside greenways [52]. For croplands, the arable land redline policy should be strictly followed. Then, for large areas of sloping croplands that are unsuitable for agriculture, the project of returning farmland to forestland and grassland should be conducted to improve the ecological benefits, while for croplands with more favorable conditions, farmland of a high standard should be developed, and intensified production should be performed. Areas with low LU degrees and low ESVs should be used as ecological restoration areas. For example, ecological restoration should be conducted in bare-soil areas with severe erosion, and in the low- and medium-coverage grasslands in the shallow mountainous areas of the loess hills, grazing should be prohibited and grassland conservation and restoration should be performed to prevent soil erosion. Areas with high LU degrees and high ESVs in the basin could be used as ecological transition zones to further increase eco-friendly LU types, to expand the ecological benefits.

As the most significant ecologically fragile region in the world, the Qinghai–Tibet Plateau has scarce available land resources and there is a prominent tension between humans and the land [57]. In recent decades, climate change, overgrazing, urbanization, and tourism have changed the LU structure of the Qinghai–Tibet Plateau, and the ecological environment has been severely affected [1]. In recent years, scholars have successively conducted research on the interaction between LUCC and ESV in the Sanjiangyuan areas [40], Qinghai Lake Basin [58], and Lhasa River Basin [59], which are of great significance to explain the dynamic mechanism of the LUCC and ESV. The Huangshui River Basin is the basin with the largest population density in the Qinghai–Tibet Plateau. According to comparisons with other areas in the Qinghai–Tibet Plateau, the LU pattern and ESV of the Huangshui River Basin have been greatly affected by human activities; the diversified ecosystem in the basin contains very high ecosystem service values and there is a significant negative spatial correlation between LU and ESV. Therefore, the conflict between human beings and the land during the process of sustainable development of the basin is still prominent. In addition, forestland and grassland are the main areas of the ESV in the Qinghai–Tibet Plateau. Therefore, the continuous implementation of ecological protection and restoration projects, including Grassland Restoration, the Grain for Green Program, and the Sanjiangyuan Ecological Protection and Construction Project can offer considerable ecological benefits and ecological values for the Qinghai–Tibet Plateau. Lhasa River Basin is the second-largest population-gathering area in the Qinghai–Tibet Plateau [1,2]. At present, there have been many problems in the Lhasa River Basin, such as a decline in ecological service function that is caused by grassland degradation, the spatial autocorrelation pattern of forestland and grassland being greatly affected by the change in LU mode [59]. As the area of the Lhasa River Basin is twice as large as that of the Huangshui River Basin, forestland and grassland are still the main contributors to ESV in this basin. Therefore, sufficient attention should be paid to this basin, and the spatial autocorrelation pattern analysis of LU and ESV and a study of the relationship between them should be carried out [59]. This study can provide reference material for the formulation of ecological protection policies and urban landscape planning in the Lhasa River Basin and can also provide some reference material for other developing countries, such as Egypt [60] and India [61], which are also facing ecological and environmental problems.

## 6. Conclusions

The Huangshui River Basin is one of the most densely populated areas in the Qinghai–Tibet Plateau and is also one of the most important sub-basins in the upper reaches of the Yellow River. The ecological services of biodiversity protection, water conservation, food production, and other functions are highly important for regional development. In this study, we employed land-use data obtained from GEE and combined it with an ESV evaluation method at the grid scale to analyze the spatial correlation and interactions between LU and ESV in the Huangshui River Basin. The results of this study can guide the river-basin planning and ecological-service-function improvement of the Huangshui River Basin. The main conclusions are as follows:

(1) With the support of the GEE cloud platform, in combination with Sentinel-1/2 active and passive remote-sensing data and other auxiliary classification features, the gradient tree-boosting ensemble learning classifier was used to efficiently obtain LU data with a spatial resolution of 10 m for the Huangshui River Basin in 2020. The OA reached 88% and the K coefficient was 0.86, which indicated that the comprehensive application of cloud computing, multisensor data, and ensemble learning can generate relatively accurate LU data.
(2) The LU types in the Huangshui River Basin showed significant positive spatial autocorrelations and the spatial agglomeration of cropland was the strongest. The LU degree in the basin also had a strong positive spatial correlation, while the LU degree in urban and agricultural areas showed H-H agglomerations. The basin ESV exhibited a significant positive spatial correlation and areas with high ESVs were woodlands, grasslands, reservoirs, and wetlands, showing H-H agglomerations.
(3) There was a significant negative spatial correlation between the LU degree and ESV in the Huangshui River Basin, and the enhancement of the LU degree in the basin could cause negative spatial spillover effects to the ESV of the surrounding areas.

This study had the following shortcomings: first, due to the complex topography of the study area, the impact of topographic factors on the pattern of the spatial autocorrelation of LU in the basin needs to be studied in depth. Second, being limited by the spatial resolution of remote sensing images, the accuracy of the ESV assessment was also limited. The use of images with a higher spatial resolution to generate LU data with better accuracy will be considered in subsequent studies to improve the ESV assessment accuracy. Third, the ESV within each grid, obtained using the grid method, was relatively static (the ESV and LU between the actual adjacent grids were mutually influential), and the composition of LU or ecosystem services in the surrounding grid should also be considered when conducting an ESV assessment based on a grid.

## Figures and Tables

**Table 1:** Image classification characteristics.

Type	Classification Features	Description	References
Spectral bands	Blue band Green band Red band NIR band	Using the 2nd, 3rd, 4th, and 8th bands of the Sentinel-2 MSI data for calculation, with a spatial resolution of 10 m	[30]
Spectral indices	Normalized differential vegetation index (NDVI) Normalized differential water index (NDWI) Ratio resident-area index (RRI)	Calculated from the Sentinel-2 MSI data, with the enhancement of vegetation, water bodies, and urban and rural industrial/mining/residential lands. Spatial resolution is 10 m	[31,32,33]
Texture information	Contrast Variance Mean Entropy	After performing the principal component analysis (PCA) on the 2nd, 3rd, 4th, and 8th bands of the Sentinel-2 MSI data, the first principal component was used to calculate the gray-level cooccurrence matrix (GLCM) to reflect the information on the distance, grayscale level, and direction in the image. Spatial resolution is 10 m	[34]
Terrain information	DEM Slope Aspect Hill shade	The 12.5 m ALOS DEM data, which mainly display the topographic information, were used for the calculation, and finally resampled to 10 m	[34]
Polarization bands	VV + VH polarization data for ascending and descending orbits	Sentinel-1 SAR data were used to extract the surface-scattering characteristics. Spatial resolution is 10 m	[35]
Tasseled cap changes	Brightness Greenness Wetness	The 3rd, 4th, and 8th bands in the Sentinel-2 MSI data, which mainly reflect the moisture and brightness of soil and vegetation, were used for calculation. Spatial resolution is 10 m	[36]

**Table 2:** ESV equivalent per unit area in the Huangshui River Basin.

Ecosystem Service Functions		Cropland	Forestland	Other Forestland	High-Coverage Grassland	Medium-Coverage Grassland	Low-Coverage Grassland	Water	Wetland	Unutilized Land
Supply	Food production	0.85	0.27	0.19	0.23	0.00	0.00	0.80	0.51	0.01
	Raw material production	0.40	0.63	0.43	0.34	0.29	0.20	0.23	0.50	0.02
	Water supply	0.02	0.33	0.22	0.19	0.16	0.00	8.29	2.59	0.01
Regulation	Gas regulation	0.67	2.07	1.41	1.21	1.03	0.73	0.77	1.90	0.07
	Climate regulation	0.36	6.20	4.23	3.19	2.71	1.91	2.29	3.60	0.05
	Environment purification	0.10	1.80	1.28	1.05	0.89	0.63	5.55	3.60	0.21
	Hydrological regulation	0.27	3.86	3.35	2.34	1.99	1.40	102.24	24.23	0.12
	Soil conservation	1.03	2.52	1.72	1.47	1.25	0.00	0.93	2.31	0.08
Support	Nutrient cycling	0.12	0.19	0.13	0.11	0.00	0.00	0.07	0.18	0.01
	Biodiversity	0.13	2.30	1.57	1.34	1.14	0.80	2.55	7.87	0.07
Culture	Aesthetic landscape	0.06	1.01	0.69	0.59	0.50	0.35	1.89	4.73	0.03

**Table 3:** Significance test for global Moran’s *I* index of each LU type.

Index	Urban	Crop Land	Forest Land	Other Forest Land	High-Coverage Grassland	Medium-Coverage Grassland	Low-Coverage Grassland	Water	Wetland	Unutilized Land
Moran’s *I*	0.76	0.89	0.65	0.84	0.85	0.87	0.77	0.40	0.57	0.75
Z score	137.40	152.87	117.90	150.88	150.98	159.95	135.70	73.15	108.25	132.06
*p*	<0.001									

**Table 4:** ESV values of various LU types in the Huangshui River Basin.

Type	Cropland	Forest Land	Other Forest Land	High-Coverage Grassland	Medium-Coverage Grassland	Low-Coverage Grassland	Water	Wetland	Unutilized Land	Total
Value/CNY billion	3.55	0.70	13.63	7.54	3.95	0.95	2.19	0.60	0.07	33.18
Value percentage/%	10.71	2.11	41.09	22.73	11.90	2.85	6.60	1.81	0.20	100.00

## Data Availability

The data presented in this study are available upon request from the first author.
